# "COVID-19 spreads round the planet, and so do paranoid thoughts". A qualitative investigation into personal experiences of psychosis during the COVID-19 pandemic

**DOI:** 10.1007/s12144-021-02369-0

**Published:** 2021-10-12

**Authors:** Minna Lyons, Ellen Bootes, Gayle Brewer, Katie Stratton, Luna Centifanti

**Affiliations:** 1 School of Psychology, Liverpool John Moores University, Tom Reilly Building, Byrom Street, Liverpool, L3 3AF UK; 2 Department of Psychology, The University of Liverpool, Bedford Street South, Liverpool, L69 7ZA UK

**Keywords:** Psychosis, Pandemic, COVID-19, Mental health, Discussion forum, Reddit, Thematic analysis

## Abstract

The COVID-19 pandemic is likely to affect people who have had previous experiences of psychosis – either positively or negatively. A research gap exists in looking at qualitative experiences of the pandemic. In the present study, we address the research gap in those who self-identified as having psychosis via Reddit discussion forum posts, collecting data from a popular online community. Sixty-five posts were analysed using inductive thematic analysis. Five overarching themes were identifie; declining mental health, changed psychosis experiences, personal coping experiences, social connectedness and disconnectedness, and COVID-19 as a metaphor. The data show that there are varied experiences associated with the pandemic. People who have experiences of psychosis do not only have vulnerabilities but may also perceive themselves as having strengths that allow them to cope better.

## Introduction

The COVID-19 pandemic is likely to pose significant additional psychosocial stressors to individuals with severe mental health difficulties (Brooks et al., 2020; Druss, 2020), including psychosis (Zhand & Joober, 2021). This could lead to exacerbation of the symptoms of psychosis for some, increasing mental distress (Hamada & Fan, 2020; Kozloff et al., 2020). The pandemic-related pressures are likely to have a relationship with multiple factors, such as fear of the virus, social isolation measures, and lack of appropriate care (Gillard et al., 2021; Kozloff et al., 2020; Zhand & Joober, 2021). For example, Nicole Kozloff et al. (2020) discussed how social distancing measures diminish social support/casual contacts/formal help, all of which are crucial in supporting individuals with psychosis. The social distancing measures could drastically worsen mental wellbeing, putting people at an increased risk of suicide (Kozloff et al., 2020). Other identified pandemic burdens could be in the overload of information regarding the virus (i.e., the “infodemic”), which could worsen hallucinations and delusions (Hamada & Fan, 2020). There is now a wealth of both qualitative and quantitative investigations on how the pandemic has affected people with pre-existing mental health conditions in various countries (e.g., Brewer et al., 2021; González-Blanco et al., 2020; Pinkham et al., 2020; Tandt et al., 2021). However, few studies have reported the experiences of people with psychosis at the beginning of the first pandemic lockdown in Spring 2020.

Overall, empirical investigations on how virus epidemics/pandemics influence individuals with psychosis/schizophrenia are sparse, and the findings are inconsistent. In a systematic review of previous virus epidemics, Brown et al. (2020) found that psychosis experiences related to increased anxiety, higher risk perception, and increased interpretation of the virus as a meaningful sign. However, previous virus epidemics are not directly comparable to the current pandemic. In a sample from Spain, González-Blanco et al. (2020) discovered that participants with severe mental health disorders (including psychosis) had similar levels of distress during the COVID-19 pandemic as participants with common mental health disorders, both of whom reported more anxiety than healthy controls. A longitudinal survey from the U.S. suggested individuals with a schizophrenia diagnosis may even be resilient to the adverse psychological effects of the pandemic, at least at the early stages of the virus outbreak (Pinkham et al., 2020). Finally, in a qualitative study in the UK, Gillard et al. (2021) discovered that lack of social connectedness and access to care were some of the most prominent sources of difficulty in a diverse sample of people with psychosis. However, some participants made new connections with their neighbours, as well as with online communities. It is clear, therefore, that the pandemic could relate to multiple experiences, both negative and positive.

To study the experiences of people with psychosis during the first COVID-19 lockdown, we chose a novel method – public discussion forums. Internet discussion forums have become a popular tool for peer-support for a diverse range of experiences, including severe mental health conditions. These forums provide an online community where people can ask questions and share their lives with like-minded peers. Research has already analysed discussion forums to understand how pandemic relates to opioid use (Krawczyk et al., 2021), specific health (Brewer & Stratton, 2020) and mental health (Brewer et al., 2021) conditions, and intimate partner violence (Lyons & Brewer, 2021). In addition, discussion forums have been used in mental health research into experiences of a first episode of psychosis (Spikol & Murphy, 2019), and the use of language in psychosis (Lyons et al., 2018). We decided to use a constructivist, descriptive approach, conducting an inductive thematic analysis to allow the data to speak for the participants’ personal experiences with the pandemic.

## Methods

### Selection of Forum Posts

Reddit is a discussion forum platform, containing over 10,000 “subreddits” that are generated by users in order to discuss topics of common interest (Widman, 2020). Reddit has been used by research communities to investigate a range of sensitive subject areas. We chose a popular subreddit that has more than 16,600 users who have experience of psychosis. We selected posts where people were discussing their own, personal experiences, or asking for advice for themselves (rather than for other people). In order to find posts that related to the pandemic, we searched posts using words “COVID, corona, virus, and pandemic”, written between 1st March, 2020-1st June, 2020. After entering each search term, we went through the threads, excluding those that focused on the experiences of someone else, offered advice without sharing personal experiences, or did not mention the COVID-19 pandemic. Each username (“user”) was analysed as one unit. We terminated data collection when all the relevant posts were collected, obtaining 65 in total.

### Ethical Issues

The study was approved by the Institutional Review Board (ref: 7680). When designing and conducting the study and reporting our findings we consulted ethical guidelines, previously published research, and available guides (e.g., Smedley & Coulson, 2021). In particular, we considered the public or private nature of the information shared, the potential for benefit or harm, and the feasibility of seeking informed consent (Eysenbach & Till, 2001; Roberts, 2015). For example, users may feel distressed about their posts being analysed for a scientific study without their consent. However, seeking consent is not practical for several reasons (e.g., Smedley & Coulson, 2021). In addition, due to large number of members in the subreddit, we considered that the posts were more “public” than “private” in nature, as they were addressed to a large number of online strangers (Eysenbach & Till, 2001).

As researchers, we are fully aware that the posts contain sensitive information, and the forum users are not aware that their discussions are used for research. However, our aim is not to analyse personal characteristics of individuals, but to gain a broader understanding of the issues that affect people who self-identify as experiencing psychosis during the pandemic. We hope that the practical value of this under-researched area outweighs the potential harms.

In mitigating potential harm, we adopted a number of measures in accordance with professional body guidelines (e.g., BPS, 2017) and advice from discussion forum researchers (Smedley & Coulson, 2021) to protect the anonymity of the forum users. For example, we do not reveal online usernames, and have allocated a number to each username to preserve that anonymity. In addition, we altered the wording of the quotes, and entered the quotes in google search engine and the subreddit site to make sure the quote cannot be traced back.

### Data Analysis

The research team consisted of four researchers (three faculty members and one Master’s student). The team has extensive experience in discussion forum research; studies on psychosis/mental distress; and qualitative coding/thematic analysis. Two researchers (ML and EB) independently coded the data for reflexive inductive thematic analysis (Braun & Clarke, 2021). They read the posts several times, established codes independently, and discussed the codes and themes. The codes were then organised into themes, which were discussed and agreed with the wider research team. We applied Leininger’s (1994) six criteria (credibility, confirmability, meaning in context, recurrent patterning, saturation, and transferability) when assessing the trustworthiness of our findings.

**Credibility** refers to how true, believable, and accurate the data are. Discussion forum posts can have high credibility because they are anonymous, and the content is free of researcher biases (i.e., prompted by the users themselves). On the other hand, the credibility is somewhat compromised by the lack of participant involvement in analysing the data. In order to overcome this, we discussed our interpretations of the findings extensively, acknowledging our potential biases, and attempting to adopt the perspective of the forum users. **Confirmability** is achieved by confirming the findings with the participants. At times, the posts in this study would have benefited from further questions. However, we believe that even without the confirmability, the posts are an honest reflection of the individuals’ experiences. **Meaning-in-context** is about understanding the findings in the context that they occur in. Our interpretations of the data are compatible only within the specific context of COVID-19 pandemic. **Recurrent patterning** refers to analysing “stories” that have designated patterns within the data. Our posts often consisted of similar sequences across the posters (e.g., several pandemic stressors increasing distress). **Saturation** refers to termination of data collection/analysis at the point when no new information is forthcoming from the informants (although see Braun & Clarke, 2021, for critique in applying saturation to thematic analysis). In our data, we did feel that no new codes/themes were emerging beyond the posts that we collected. **Transferability** is about the ability to transfer the findings to another similar context. Our findings did mirror other studies investigating the experiences of individuals during the pandemic (e.g., Brewer et al., 2021; Gillard et al., 2021).

## Results

We identified five themes: (i) declining mental health (subthemes: general poor mental health; suicidal ideation; stress; sleep issues), (ii) changed psychosis experiences (subthemes: increased psychosis; relapse; COVID-19 related hallucinations; fear of contamination; personal causation of COVID-19; conspiracy theories and fear of government), (iii) Personal positive and negative coping experiences (subthemes: medication; engaging with the voices and hallucinations; prior experience), (iv) social connectedness and disconnectedness (subthemes: social isolation; availability of social support; sharing experiences online), and (v) COVID-19 as a metaphor (subthemes: explaining psychosis to others; reflective behaviour).

## Theme 1: Declining Mental Health

Forum users often reported that their overall mental health had deteriorated due to the pandemic. They experienced a range of difficulties, from sleep disturbance to suicidal ideation.

### General Poor Mental Health

Poor mental health was discussed widely, with an emphasis on how mental health had deteriorated due to the pandemic. One user reported: “*I’ve been spiralling down, and my mental health has gotten detrimentally worse*” (Post 2). A common mental health difficulty was depression, for example, “*no energy to even get off the couch*” (P48); “*I’ve been depressed because of the pandemic… I’m slipping back into the dark place*” (P43). Declining mental health had been described as a daily struggle by one user, “*I’m now having daily breakdowns*” (P8).

### Suicidal Ideation

Many also talked about suicidal ideation. One user described a recent suicide attempt because of the pandemic, “*three weeks ago, I lost control of myself and I almost killed myself, and I barely remember it*” (P3); another reported the escalation of suicidal voices, with the voices being “*back and stronger*” (P4). Suicide was also perceived as a means to escape the suffering of the pandemic, “*by killing myself I would ascend to a greater plane of life where there is no daily suffering*” (P30).

### Stress

Many accounts reported that levels of stress had increased exponentially during the pandemic. For many, the stress exacerbated psychosis, “*the stress compounded, and my symptoms returned... the stress my mind goes through is true suffering*” (P44). Other posts demonstrated an intersection between mental and physical health, “ *I experienced hallucinations and delusions due to pandemic stress as an asthmatic*” (P11); and *“a huge period of stress has been building up… COVID-19 has really messed me up*” (P15).

### Sleep Issues

Users also suffered with sleep issues due to the pandemic, either sleeping too much or too little. One stated, “*sleep is incredibly important for my diagnosis, but I’m refusing to go to bed*” (P42). Others talked about “*I’ve been oversleeping, I sleep for over ten hours a day now*” (P50); “ *I sleep almost till noon*” (P52); and “*sleep is all over the place, twelve hours one night and four hours the next, then up for a day or two*” (P30).

## Theme 2: Changed Psychosis Experiences

Many discussed a novel set of experiences influenced by the pandemic. For example, forum posts described an intensification of experiences during the pandemic and experiencing COVID-related psychosis.

### Increased Psychotic Experiences

The pandemic appeared to intensify experiences of psychosis. Some individuals felt their psychosis experiences were more frequent and became more intense. “*full-blown seeing things… COVID has really messed me up*” (P14); “*it’s been hard to tell whether the increased frequency of voices is due to isolation or if that part of my disorder is still developing*” (P15); “*due to the social isolation I’m getting further immersed in my own world*” (P25).

### Relapse

The pandemic also brought a risk of relapse, for example “*my schizoaffective symptoms have returned*” (P44); “*many dark feelings are back*” (P36); and “*I haven’t experienced psychosis in years, not like this*” (P14). Others felt they were on the path to relapse but had not yet reached it, “*I haven’t had an episode in a couple years I think but I’m afraid I can feel one coming on*” (P6); “*I can already feel myself slipping*” (P20); and “*I’m terrified of my paranoia coming back*” (P22). Thus, even if symptoms had not returned or intensified, there was anxiety around the possibility.

### COVID-19 Related Hallucinations

The pandemic appeared to alter the psychotic experiences of participants, resulting in novel, disease-themed experiences. One participant had begun to “*see bugs crawling on my walls and furniture… enough to make me scream as I am terrified of bugs or enough to make me get up and look only to find nothing*” (P50). One possibility is this is a visual representation of the virus, rooting itself in existing fears and compounding the current psychotic experiences. Another described a hallucination where they “*regularly see a plague doctor who was never here before, but he’s here now. And he seems to be willing to go away once COVID is over*” (P10).

### Fear of Contamination

Participants reported an intense fear of contracting the virus and contamination, expressing itself as an increased paranoia. One user described how their previous occupation in a laboratory had impacted their psychosis, “*I had a phobia that I would get infected with HIV and transmit it to others... obsessing on catching COVID-19 and accidentally infecting people*” (P36). Others wrote how the fear of contaminating others was greater than the fear of becoming infected, “*I had a cold that turned into a sinus infection, terrified I had COVID-19 and would infect people*” (P56).

### Personal Causation of COVID-19

One of the notable changes was a new belief that user caused the pandemic. This belief was often communicated through voices, for example “*…they have been saying that everything is my fault - the virus, the climate emergency, the general state of politics*” (P29); “*…been hearing different voices, constantly told this is my fault*” (P31). Often, the voices were saying that the pandemic was a punishment for disobeying their command, “*I’m terrified of [the voices] coming back to tell me COVID-19 is my fault for disobeying them*” (P22); “*I think this whole COVID-19 is happening because I didn’t believe in Jesus when I died*” (P45). Whilst some participants did not disclose whether they heard voices blaming them for the pandemic, they still believed they were responsible, “*even if my thoughts weren’t the initial cause, they were somehow connected to it*” (P37); “*I caused COVID-19... and I don’t know what to do to make this stop*” (P38); “*I feel like I am going to be put to death... that the virus and the economy is my fault*” (P54).

### Conspiracy Theories and Fear of Government

Many users expressed distrust of their governments, describing their own theories for government involvement, “*the rich can afford ventilators and health care. It is starting to build a government conspiracy narrative*” (P56); and “*I do believe the US government would use bioweapons to take out their “enemies”... after all it did start in China and they were having [issues with China]*“ (P59). Others felt their government had the potential to harm them in conjunction with the pandemic, “*the government’s out to get me because of what I know, and if they do I know something bad is going to happen*“ (P29); and “*the government is… making it worse*“ (P41). Conspiratorial beliefs also included non-governmental theories regarding the source of the pandemic, “*for me the virus plays into my Truman show delusion. It makes me think the virus was made up solely for drama and to kill off excess characters they didn’t need anymore*” (P51).

## Theme 3: Personal Negative and Positive Coping Experiences

Users displayed an assortment of coping methods with both positive and negative connotations attached to them.

### Medication

Medication was one of the most widely used coping mechanisms with varying degrees of success. For some, medication resulted in positive experience, “*I increased my meds which seemed to help*” (P2). Others had limited success of using medication, for example, “*I found myself having to take Ativan more often in one week than I had all of last year… I’m so scared of developing Ativan dependency*” (P44). Other recurring topics included inadequate care, “*the only treatment I got was medication and a weekly phone call*” (P52), or not having enough medication to cope effectively “*I have medication for agitation but I’m running low and my doctor won’t refill them yet*” (P26).

### Engaging with the Voices and Hallucinations

Engaging with the voices was a unique coping mechanism. One user described how “*since the lockdown I’ve been talking to the voices in my head. It’s easier than stressing about them*” (P9). Another outlined how they coped with hallucinations by engaging directly with them, “*I have made the hallucinations my “friends” which is a negative thing to do but they themselves aren’t as bad*” (P3). Many others found the voices too distressing to engage with, and subsequently did not use them as a coping mechanism.

### Prior Experience

Prior experience of psychosis led to some describing feeling prepared for the pandemic. Some noted how isolation is an experience they have already lived through, “*being in quarantine is nothing new”* (P16); “*I’ve come to realise during this pandemic that social distancing and self-isolation is just a normal day for me*” (P46). Others discussed how their psychosis has been more distressing than the pandemic, “*I thank my psychosis as it really did prepare me for everything that is happening*” (P18); “*I went through the paranoia already and I am not even the slightest bit scared this time round*” (P19); “*maybe because I have been through hell myself… what’s the big deal?*” (P17).

## Theme 4: Social Connectedness and Disconnectedness

This theme had subthemes relating to the pandemic causing social disconnection, but also how some of the social connections were strengthened during this period.

### Social Isolation

Across the posts, there were multiple examples of social isolation. Some had a few close individuals to rely on, “*I stay in my room and talk to no one except my parents who I live with*” (P50). Others found themselves totally socially isolated, “*no support system just me alone*” (P35); “*barely anyone to talk to as normal*” (P46); “*I just don’t have anywhere to go nor talk to anyone*” (P47). A few described how their social circles had collapsed due to the pandemic, “*my significant other packs up and leaves and now I’ve been told to go back into isolation*” (P20); “*my entire coping strategy has collapsed with this virus - gym, martial arts, socializing*” (P49). Many felt that due to social isolation specifically, their mental health had deteriorated and their experiences of psychosis had escalated. For example, “*due to the social isolation, I’m getting further immersed in my own world*” (P25); “*isolation from corona is making me feel even worse... I feel like I’m going to burst*” (P64); “*ever since COVID-19 started I’ve felt super lonely...”* (P43); *“I live alone... self-isolation is dangerous after a couple days for me*” (P33).

### Availability of Social Support

Lack of formal support from the healthcare system was an issue that resonated with many “*none of my therapists are seeing anyone as they’re suspected infected*” (P20). The perceived lack of support led to service user disengagement. The informal social support available was especially important, “*at least a lot of people, including my direct supervisor at work, have been supportive and understanding*” (P44). Some even described how their friends were willing to support them even if it meant breaking rules “*I had to break the rules and ask someone to come over to stay with* me” (P14); “*one of my friends is nice enough to still see me in person, but I’m constantly shamed for it by other people*” (P33).

### Sharing Experiences Online

Discussion forums were used as a platform to share experiences, and as a space to offload feelings that the users were unable to express offline. Users described how they “*panicked and I had to tell someone*” (P61); “*needed to get some stuff off my chest*” (P30). They also used the discussion forum to learn how other people living with psychosis were coping with the pandemic, enquiring about coping mechanisms and similar experiences; “*do you have any tips on how to cope and avoid being so paranoid about it*?” (P1), and “*anyone else experience anything similar?*” (P10).

## Theme 5: COVID-19 as a Metaphor

The COVID-19 was used as a vehicle to help others understand psychosis. Users also described how current societal behaviours mimic their previous psychotic behaviours, finding this useful in validating experiences.

### Explaining Psychosis

Pandemic was used as a metaphor to help others understand what psychotic experiences are like, “*the COVID situation is perfect for explaining paranoia to people... the things I’m paranoid about are real to me too. The pandemic feeling is just like what it’s like to be me*” (P40), and “*COVID-19 spreads round the planet, and so do paranoid thoughts* …*. Where is this virus, how can I stop it*… *scared and alone fighting a real ghost that no one can see*” (P41).

### Reflective Behaviours

Reflective behaviours are those behaviours associated with psychosis, seen in a general population. User 40 described how “*I see so many people mirroring the behaviour I have when I’m in an episode*”, referring to others constantly researching topics causing their paranoia. These reflective behaviours appeared to validate the experiences of those living with psychosis, providing a degree of comfort, “*it’s good that this virus has non-psychotic people say things I normally got called “delusional” for*” (P55). Users also found new behaviours in others to be gratifying. “*I have sickness delusions…now when everybody is freaking out, I somehow almost feel better… an “I told you so” moment*” (P61); and “*people finally wash their hands, so lovely*” (P61).

## Discussion

This study utilised an inductive thematic analysis in understanding personal experiences at the beginning of the COVID-19 outbreak, revealing a range of pandemic-related experiences in discussion forum users who self-identified as experiencing psychosis. We constructed five overlapping themes around declining mental health, changed psychosis experiences, personal coping, social connectedness and disconnectedness, and COVID-19 as a metaphor. It was clear that in addition to increased challenges, psychosis also related to resilience in dealing with the pandemic.

Many reported a decline in mental health, including changes in psychosis-related experiences. This is consistent with suggestions in recent theoretical papers, outlining how COVID-19 related factors (e.g., social distancing, loneliness, fear of contamination, overload of information) can lead to exacerbated distress, delusions, and hallucinations in individuals with severe mental disorders (Hamada & Fan, 2020; Zhand & Joober, 2021). The impact of reduced social connections due to social distancing and lockdown measures seemed to play a substantial role in worsening mental health. Social support and connections with other people are major factors increasing resilience during the pandemic (Killgore et al., 2020), and social isolation can be especially harmful for those who experience psychosis (Badcock et al., 2020; Gillard et al., 2021). Online communities have become a crucial source of social support during the pandemic (e.g., Brewer et al., 2021), and our results demonstrate that online discussion forums are useful outlet for those who have psychosis.

Worsening mental health was also evidenced in delusions, and a change in the hallucinations and voice content. Many users held themselves responsible for the pandemic, consistent with self-blaming attributional biases found in psychosis (Langdon et al., 2013). The self-blame was reflected in the content of the voices, accusing the individual of causing the pandemic. Some discussed how their hallucinations had changed in line with the pandemic, including visions related to death and contamination. Research has found that hallucination and voice content sometimes match with traumatic experiences of the individual (Peach et al., 2020). It is evident that many of the discussion forum users experienced the pandemic as a traumatising event.

In addition to change in psychotic experiences, the forum users also demonstrated a distrust of governments, and/or discussed COVID-related conspiracy theories. These findings complement the quantitative research that suggests a link between schizotypy, paranoia, and COVID-conspiracies in the general population (Ferreira et al., 2020; Kuhn et al., 2021; Larsen et al., 2021). Studies in diverse samples have suggested that belief in conspiracy theories relates to worsening of mental health (e.g., Chen et al., 2020; Leibovitz et al., 2021). It is possible that the forum users in the present study experienced worsened mental health partially due to information overload and conspiracy beliefs (see also Hamada & Fan, 2020). Conspiracy beliefs have large public health consequences, impeding the attempts to control the spread of COVID, and influencing the success of vaccination programmes (e.g., Romer & Jamieson, 2020). It would be useful to incorporate COVID-19 specific conspiracy theory meta-cognitive training in existing therapies (see Kumar et al., 2015). At an individual level, this could allow people to challenge their conspiratorial beliefs, leading to better containment of the pandemic at a societal level.

Not all posts reported worsened mental health, but many also reflected strengths of the individuals. Indeed, having already experienced severe mental distress seemed to be a protective factor, helping people to cope better (see also Burton et al., preprint). Prior lifetime adversity could increase resilience in the face of new hostile events (Seery et al., 2010), and experiences with psychosis seem like a good fit with the extraordinary, new circumstances of the pandemic. Many also discussed the pandemic as a useful metaphor for explaining psychosis to others. Previous research has shown that simulating psychosis-like experiences increases compassion and understanding (Riches et al., 2018), and that explaining psychosis to others can reduce self-stigma (Huggett et al., 2018). The psychosis-like behaviours of the general population, such as increased isolation and fear of unseen viruses, seemed like a source of comfort. The pandemic may have the unexpected positive consequences in reducing self and other-stigma and increasing understanding around the psychotic experiences.

The resilience was also evident in some of the coping strategies employed by the discussion forum users. They talked about different methods for dealing with psychosis, including medication, and engagement with the voices and hallucinations. For some, medication was necessary, and for others, it was a cause of additional worry. Engagement with the voices and hallucinations was a predominantly positive experience. These findings bring additional support to the Making Sense of Voices-approach to therapy (Steel et al., 2020), which advocates engagement and understanding of the voices rather than dampening them with medication. Psychological interventions such as Talking with Voices therapy (Longden et al., 2021) could be useful for increasing resilience, and in helping people to navigate through the new, complex circumstances related to the global pandemic.

## Limitations and Implications

There are some limitations with the study. First, although the posts provide a valuable insight into the first lockdown issues that were most pertinent to those with psychosis, it was not possible to confirm the location nor the demographics of the users. Reddit users are mostly from the U.S. (Clement, 2021), and we cannot speculate what the experiences are like in more diverse samples outside the Reddit communities. There are significant cross-country differences in COVID-19 experiences and mental health outcomes (e.g., Geirdal et al., 2021; Terraneo et al., 2021; Wang et al., 2020), as well as cultural and country-differences in experiences of psychosis (Lyons et al., 2020; Wüsten & Lincoln, 2017; Wüsten et al., 2018). It would be interesting to compare psychosis and the lived experiences of the first lockdown in individuals from different countries and cultures.

Second, the themes that we constructed had some overlap with each other, which is not ideal (Braun & Clarke, 2021). For example, declining mental health was clearly connected with most of the other themes (e.g., changed psychosis experiences, personal coping experiences, social connectedness and disconnectedness). However, our themes formed coherent, interpretive, creative stories (see Braun & Clarke, 2019), which made sense in the complex new environments produced by the pandemic.

Our findings have some implications for treatment and support. It is obvious that individuals with psychosis are especially vulnerable to disruptions in social contacts, which was an inevitable side effect of the first COVID-19 lockdown. In the future, it may be beneficial to develop internet-based interventions that are aimed to increase social connectedness, for example, in the form of social networking with peers (Alvarez-Jimenez et al., 2013). It is essential that access to formal services i msaintained (e.g., tele-therapy, Wood et al., 2021), and that individuals are supported to understand the myriad of ways in which the pandemic may influence the experience of psychosis.

As well as being at the receiving end of therapies, people with a history of psychosis possess valuable knowledge that could be useful in helping others who do not have the same lived expertise (Florence et al., 2021). The world experienced unusual psychosis-like paranoia and erratic thinking at the start of the pandemic (e.g., Suthaharan et al., 2021; Lopes et al., 2020), with new mental health difficulties in people who did previously not have them (e.g., D’Agostino et al., 2021). Experts by experience should be employed to advise health professionals on interventions for those who have suffered as a result of the pandemic. In addition, experts by experience could advise public health campaigns and policy makers around strategies for reducing paranoia and suspiciousness associated with vaccinations and general COVID-19 health measures.

## Conclusion

There are some areas of vulnerability and new challenges for those with prior experiences of psychosis, as well as remarkable resilience to deal with the pandemic. The COVID-19 pandemic has influenced the lives of all people across the globe. In some ways, these challenges unite people in their experiences of mental distress, isolation, and searches for new coping mechanisms, which may reduce the stigma associated with psychosis. Promoting positive coping strategies and social connectedness while adhering to COVID-19 may be particularly important for future interventions.

## Data Availability

Due to the additional steps in protecting the identity of the discussion forum users, we will not make the collected posts publicly available.
